# Prognostic significance and the role in TNM stage of extranodal metastasis within regional lymph nodes station in gastric carcinoma

**DOI:** 10.18632/oncotarget.11478

**Published:** 2016-08-22

**Authors:** Xiao-Long Chen, Lin-Yong Zhao, Lian Xue, Yu-Hui Xu, Wei-Han Zhang, Kai Liu, Xin-Zu Chen, Kun Yang, Bo Zhang, Zhi-Xin Chen, Jia-Ping Chen, Zong-Guang Zhou, Jian-Kun Hu

**Affiliations:** ^1^ Department of Gastrointestinal Surgery, West China Hospital, Sichuan University, Chengdu 610041, China; ^2^ Laboratory of Gastric Cancer, State Key Laboratory of Biotherapy/Collaborative Innovation Center of Biotherapy and Cancer Center, West China Hospital, Sichuan University, Chengdu 610041, China; ^3^ West China School of Medicine, Sichuan University, Chengdu 610041, China; ^4^ Institution of Digestive Surgery, State Key Laboratory of Biotherapy/Collaborative Innovation Center of Biotherapy and Cancer Center, West China Hospital, Sichuan University, Chengdu 610041, China

**Keywords:** gastric carcinoma, extranodal metastasis, prognosis, TNM stage, C-index

## Abstract

The role of extranodal metastasis (ENM) in TNM stage in gastric carcinoma (GC) is controversial. This study was aimed to make a detailed investigation of the prognostic significance and the role in TNM stage of ENM in GC. The patients with primary GC, who underwent gastrectomy with curative intention in West China Hospital from January 2005 to December 2011, were retrospectively enrolled. The prognosis and clinicopathological traits were compared between ENM positive (ENMP) and negative (ENMN) groups in all patients, TNM I-II, III and IV stages, respectively. The significance of the number and the role in TNM stage of ENM were also assessed. In our study, 1457 patients were enrolled, with 1324 (90.9%) in ENMN group and 133 (9.1%) in ENMP group. ENMP group had significantly more advanced GC and worse prognosis (all p<0.05) than ENMN group in all patients, TNM I-II stages and TNM III stage. ENM>2 subgroup had remarkably larger tumor size (p=0.002) and more advanced N stage (p=0.016) than ENM=1-2 subgroup. The number of ENM was an independent prognostic factor in ENMP group (p=0.029). The prognosis of ENM>2 in TNM I-III stages was significantly worse than ENMN patients in TNM III stage. The C-index of TNM stage plus the number of ENM was significantly higher than that of current TNM stage alone (p=0.005). In conclusion, the patients in ENMP subgroup had more advanced GC and worse prognosis than those in ENMN subgroup. It might be more reasonable to categorize ENM>2 into TNM IV stage.

## INTRODUCTION

Gastric carcinoma (GC) is one of the most common malignancies in the world [1]. It is well known that lymph nodes are the main metastatic route of GC. Therefore, lymphadenectomy has been considered as one of the crucial procedures in surgery in order to prevent tumor recurrence and metastasis. In the postoperative pathological examination, the histology and number of lymph nodes from the specimen are the main objects. Besides the lymph nodes, some tumor nodules without histological evidence of lymph node structure within the lymphatic drainage extent may also been found, which are recorded as extranodal metastasis (ENM) with the incidence approximately 13% [2–3]. In the Japanese classification of GC according to Japanese Gastric Cancer Association (JGCA), ENM is currently recommended to be counted as a metastatic lymph node in the N determination [4]. Some studies also reported the similar result, indicating that ENM should be incorporated in N stage [5]. However, the role of ENM in TNM stage is still under debate. Some studies found that the prognosis of patients with ENM was similar to that of patients with TNM stage IV, suggesting that ENM should be categorized as M1 or N3 stage [3]. Although it is still controversial of the role of ENM, many reports showed that patients with ENM had worse prognosis than those without ENM, even in early GC [2, 5–7]. Nevertheless, the role of the number of ENM was investigated only in few studies [3]. Besides, many studies had relative small sample sizes or were only limited in lymph node positive patients [8, 9]. A detailed research of ENM in GC is still expected. The researches on ENM within regional lymph nodes station might help to establish a better staging classification system or supply the important revision suggestion to the current TNM stage of GC. The aim of this present study was to research the prognostic significance and the role in TNM stage of ENM within regional lymph nodes station in GC.

## RESULTS

**Figure 1:**
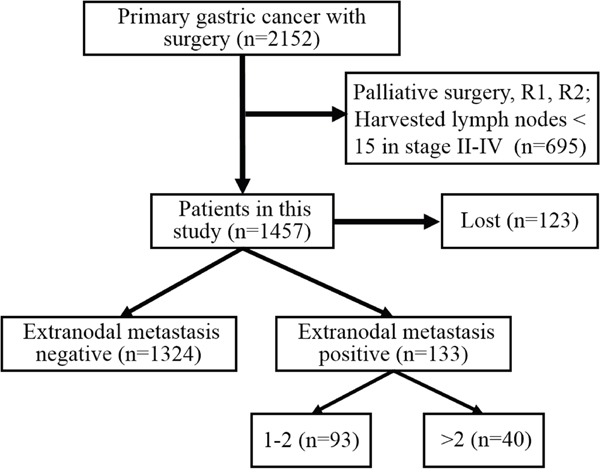
The flow chart of included patients in this study

**Figure 2:**
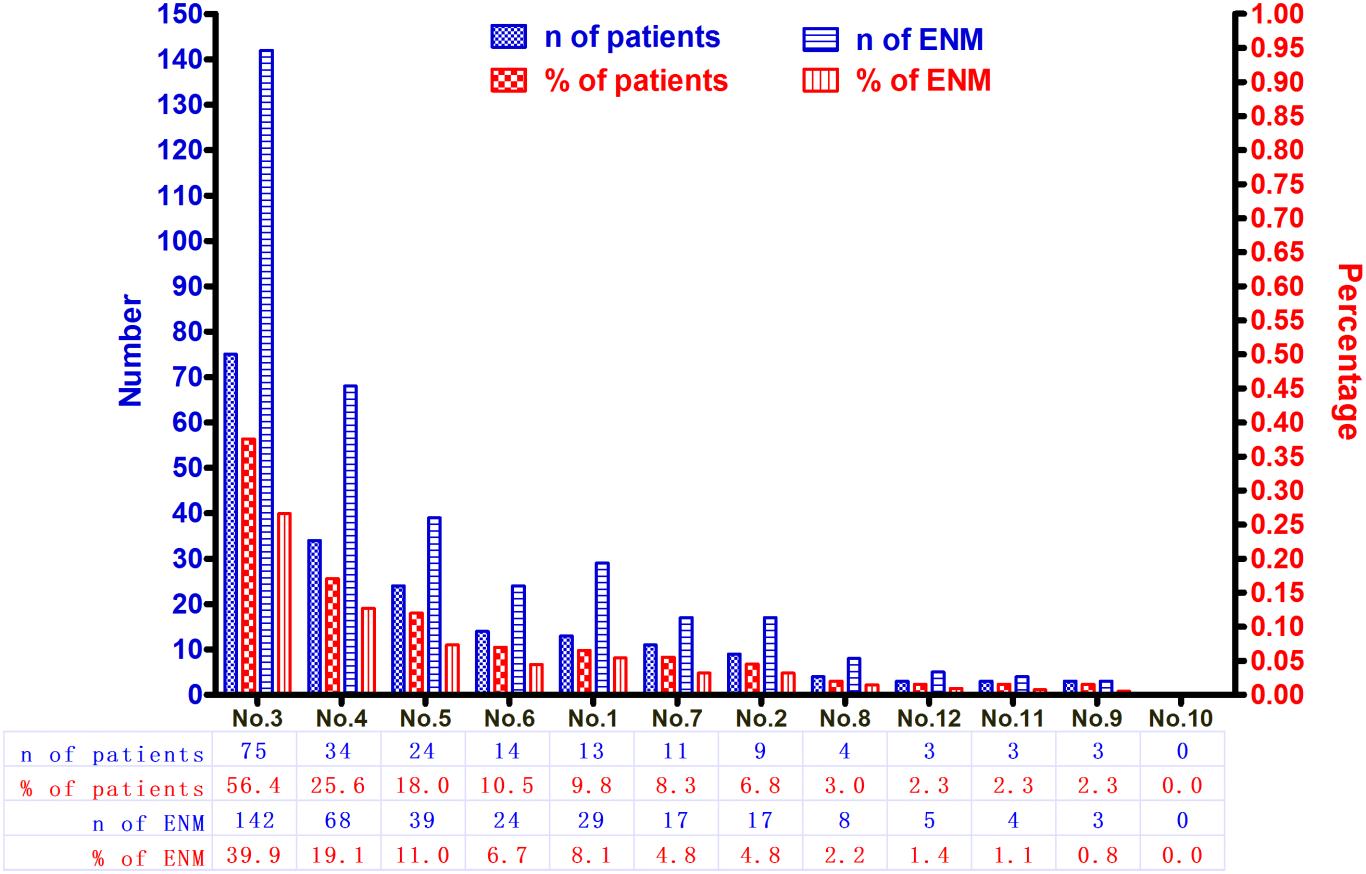
The distribution and incidence of ENM

**Figure 3:**
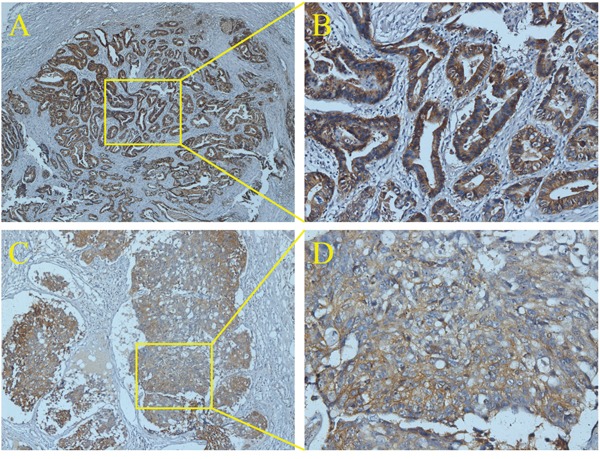
The histology of ENM by immunohistochemistry of EpCAM

In this study, 1457 patients were divided into ENM negative (ENMN) group (n=1324, 90.9%) and ENM positive (ENMP) group (n=133, 9.1%) with 356 ENM harvested. In prognosis, 1334 (91.6%) patients were followed up and analyzed. The flow chart of the patients in this study was shown in Figure 1. ENM were mainly distributed alone lesser curvature (No.3, No.5 lymph nodes) and greater curvature (No.4, No.6 lymph nodes) in more than 10% patients in ENMP group, following No.1, No.7, No.2 lymph nodes in 5%-10% patients and No.8, No.12, No.9, No.11 and No.10 in less than 5% patients (Figure 2). The distribution according to the number of ENM was similar to that according to the number of patients (Figure 2). The pathological histology of ENM in two examples was found without the histological evidence of lymph nodes structure and the cancer cells highly expressed EpCAM (Figure 3). In order to analyze the significance of ENM in different stages, the patients were subdivided into ENMN and ENMP subgroups in TNM I-II (n=679, 46.6%), III (n=699,48.0%) and IV stages (n=79, 5.4%), respectively. Regarding ENMP group, the patients were also subdivided into ENM=1-2 and ENM>2 subgroups to find out the clinical significance of the number of ENM. The clinicopathological characteristics and survival outcomes were analyzed and compared in different subgroups.

### All patients

The baseline of all patients in ENMN and ENMP groups was compared and shown in Table 1. The results showed that gender (p=0.029), longitudinal location (p=0.001), macroscopic type (p<0.001), differentiation (p<0.001), tumor size (p<0.001), vessel/nerve invasion (p=0.001), T stage (p<0.001), N stage (p<0.001), M stage (p<0.001) and TNM stage (p<0.001) were significantly different between ENMN and ENMP groups, indicating that ENMP group had more male patients and more tumors with M/UML location, Borrmann III-IV, poor differentiation, size ≥5cm, positive vessel/nerve metastasis and advanced T stage, N stage, M stage and TNM stage than ENMN group (Table 1). Logistic regression confirmed that gender (p=0.030), differentiation (p=0.011), tumor size (p=0.001), T stage (p=0.001) and N stage (p<0.001) were independently related to ENM (Table 2).

**Table 1:** Clinicopathological features of extranodal metastasis negative and positive groups in this study

Clinicopathological features		All patients (n=1457)			TNM I-II stage (n=679, 46.6%)			TNM III stage (n=699, 48.0%)			TNM IV stage (n=79, 5.4%)			ENMP (n=133, 9.1%)		
		ENMN	ENMP		ENMN	ENMP		ENMN	ENMP		ENMN	ENMP		1-2	>2	
		n=1324	n=133	P value	n=667	n=12	P value	n=594	n=105	P value	n=63	n=16	P value	n=93	n=40	P value
Age (years)	Mean±SD	56.8±11.7	58.6±11.6	0.100	56.7±12.0	65.8±11.6	0.010	57.2±11.3	56.9±11.3	0.801	54.8±11.7	64.4±9.6	0.003	58.0±12.5	59.8±9.1	0.409
	≥60	576 (43.5)	66 (49.6)	0.175	292 (43.8)	9 (75.0)	0.031	264 (44.4)	44 (41.9)	0.629	20 (31.7)	13 (81.3)	<0.001	45 (48.4)	21 (52.5)	0.664
	<60	748 (56.5)	67 (50.4)		375 (56.2)	3 (25.0)		330 (55.6)	61 (58.1)		43 (68.3)	3 (18.8)		48 (51.6)	19 (47.5)	
Gender	Male	915 (69.1)	104 (78.2)	0.029	459 (68.8)	8 (66.7)	1.000	418 (70.4)	84 (80.0)	0.043	38 (60.3)	12 (75.0)	0.277	74 (79.6)	30 (75.0)	0.558
	Female	409 (30.9)	29 (21.8)		208 (31.2)	4 (33.3)		176 (29.6)	21 (20.0)		25 (39.7)	4 (25.0)		19 (20.4)	10 (25.0)	
Longitudinal location	U	292 (22.1)	26 (19.5)	0.001	112 (16.8)	4 (33.3)	0.387	162 (27.3)	19 (18.1)	0.002	18 (28.6)	3 (18.8)	0.001	21 (22.6)	5 (12.5)	0.244
	M	273 (20.6)	32 (24.1)		120 (18.0)	1 (8.3)		137 (23.1)	28 (26.7)		16 (25.4)	3 (18.8)		22 (23.7)	10 (25.0)	
	L	741 (56.0)	66 (49.6)		432 (64.8)	7 (58.3)		286 (48.1)	50 (47.6)		23 (36.5)	9 (56.3)		46 (49.5)	20 (50.0)	
	UML	18 (1.4)	9 (6.8)		3 (0.4)	0 (0.0)		9 (1.5)	8 (7.6)		6 (9.5)	1 (6.3)		4 (4.3)	5 (12.5)	
Cross sectional location	Lesser	710 (53.6)	72 (54.1)	0.073	363 (54.4)	6 (50.0)	0.830	316 (53.2)	59 (56.2)	0.097	31 (49.2)	7 (43.8)	0.388	49 (52.7)	23 (57.5)	0.087
	Greater	118 (8.9)	12 (9.0)		72 (10.8)	2 (16.7)		42 (7.1)	10 (9.5)		4 (6.3)	0 (0.0)		12 (12.9)	0 (0.0)	
	Anterior	82 (6.2)	3 (2.3)		49 (7.3)	0 (0.0)		31 (5.2)	1 (1.0)		2 (3.2)	2 (12.5)		3 (3.2)	0 (0.0)	
	Posterior	116 (8.8)	10 (7.5)		67 (10.0)	1 (8.3)		45 (7.6)	8 (7.6)		4 (6.3)	1 (6.3)		7 (7.5)	3 (7.5)	
	Double	140 (10.6)	10 (7.5)		72 (10.8)	2 (16.7)		63 (10.6)	5 (4.8)		5 (7.9)	3 (18.8)		5 (5.4)	5 (12.5)	
	Circumference	158 (11.9)	26 (19.5)		44 (6.6)	1 (8.3)		97 (16.3)	22 (21)		17 (27)	3 (18.8)		17 (18.3)	9 (22.5)	
Macroscopic type	Early stage	212 (16.0)	2 (1.5)	<0.001	205 (30.7)	0 (0.0)	0.137	6 (1.0)	2 (1.9)	0.829	1 (1.6)	0 (0.0)	0.719	2 (2.2)	0 (0.0)	0.435
	Borrmann I	51 (3.9)	7 (5.3)		35 (5.2)	1 (8.3)		12 (2.0)	5 (4.8)		4 (6.3)	1 (6.3)		7 (7.5)	0 (0.0)	
	Borrmann II	624 (47.1)	63 (47.4)		301 (45.1)	9 (75.0)		292 (49.2)	45 (42.9)		31 (49.2)	9 (56.3)		42 (45.2)	21 (52.5)	
	Borrmann III	370 (27.9)	49 (36.8)		119 (17.8)	2 (16.7)		231 (38.9)	42 (40)		20 (31.7)	5 (31.3)		34 (36.6)	15 (37.5)	
	Borrmann IV	67 (5.1)	12 (9.0)		7 (1.0)	0 (0.0)		53 (8.9)	11 (10.5)		7 (11.1)	1 (6.3)		8 (8.6)	4 (10.0)	
Differentiation grade	Well	43 (3.2)	0 (0.0)	<0.001	43 (6.4)	0 (0.0)	0.237	0 (0.0)	0 (0.0)	0.013	0 (0.0)	0 (0.0)	0.683	0 (0.0)	0 (0.0)	0.929
	Moderately	246 (18.6)	7 (5.3)		169 (25.3)	2 (16.7)		71 (12.0)	4 (3.8)		6 (9.5)	1 (6.3)		5 (5.4)	2 (5.0)	
	Poorly	1035 (78.2)	126 (94.7)		455 (68.2)	10 (83.3)		523 (88.0)	101 (96.2)		57 (90.5)	15 (93.8)		88 (94.6)	38 (95.0)	
Tumor size (cm)	Mean±SD	4.7±2.7	6.6±2.6	<0.001	3.5±2.0	5.5±2.2	0.001	5.8±2.6	6.7±2.7	<0.001	7.1±4.0	6.5±2.6	0.555	6.1±2.5	7.8±2.7	0.001
	<2.5	237 (17.9)	1 (0.8)	<0.001	212 (31.8)	1 (8.3)	0.009	23 (3.9)	0 (0.0)	<0.001	2 (3.2)	0 (0.0)	0.593	1 (1.1)	0 (0.0)	0.002
	2.5-5	476 (36.0)	27 (20.3)		291 (43.6)	4 (33.3)		171 (28.8)	20 (19)		14 (22.2)	3 (18.8)		22 (23.7)	5 (12.5)	
	5-8	437 (33.0)	62 (46.6)		125 (18.7)	5 (41.7)		286 (48.1)	50 (47.6)		26 (41.3)	7 (43.8)		48 (51.6)	14 (35.0)	
	≥8	174 (13.1)	43 (32.3)		39 (5.8)	2 (16.7)		114 (19.2)	35 (33.3)		21 (33.3)	6 (37.5)		22 (23.7)	21 (52.5)	
Vessels/nerves invasion	Negative	1081 (81.6)	92 (69.2)	0.001	602 (90.3)	11 (91.7)	0.870	436 (73.4)	73 (69.5)	0.410	43 (68.3)	8 (50.0)	0.173	64 (68.8)	28 (70.0)	0.893
	Positive	243 (18.4)	41 (30.8)		65 (9.7)	1 (8.3)		158 (26.6)	32 (30.5)		20 (31.7)	8 (50.0)		29 (31.2)	12 (30.0)	
T stage	1a	174 (13.1)	0 (0.0)	<0.001	174 (26.1)	0 (0.0)	<0.001	0 (0.0)	0 (0.0)	0.292	0 (0.0)	0 (0.0)	0.163	0 (0.0)	0 (0.0)	0.564
	1b	157 (11.9)	0 (0.0)		156 (23.4)	0 (0.0)		0 (0.0)	0 (0.0)		1 (1.6)	0 (0.0)		0 (0.0)	0 (0.0)	
	2a	104 (7.9)	3 (2.3)		93 (13.9)	1 (8.3)		11 (1.9)	2 (1.9)		0 (0.0)	0 (0.0)		2 (2.2)	1 (2.5)	
	2b	91 (6.9)	5 (3.8)		72 (10.8)	3 (25.0)		19 (3.2)	1 (1.0)		0 (0.0)	1 (6.3)		4 (4.3)	1 (2.5)	
	3	115 (8.7)	12 (9.0)		64 (9.6)	3 (25.0)		50 (8.4)	8 (7.6)		1 (1.6)	1 (6.3)		8 (8.6)	4 (10.0)	
	4a	593 (44.8)	95 (71.4)		108 (16.2)	5 (41.7)		444 (74.7)	79 (75.2)		41 (65.1)	11 (68.8)		68 (73.1)	27 (67.5)	
	4b	90 (6.8)	18 (13.5)		0 (0.0)	0 (0.0)		70 (11.8)	15 (14.3)		20 (31.7)	3 (18.8)		11 (11.8)	7 (17.5)	
N stage	0	501 (37.8)	8 (6.0)	<0.001	494 (74.1)	8 (66.7)	0.572	5 (0.8)	0 (0.0)	0.008	2 (3.2)	0 (0.0)	0.469	6 (6.5)	2 (5.0)	0.016
	1	231 (17.4)	13 (9.8)		130 (19.5)	3 (25.0)		99 (16.7)	9 (8.6)		2 (3.2)	1 (6.3)		11 (11.8)	2 (5.0)	
	2	211 (15.9)	29 (21.8)		34 (5.1)	1 (8.3)		172 (29.0)	25 (23.8)		5 (7.9)	3 (18.8)		20 (21.5)	9 (22.5)	
	3a	251 (19.0)	56 (42.1)		8 (1.2)	0 (0.0)		220 (37.0)	50 (47.6)		23 (36.5)	6 (37.5)		45 (48.4)	11 (27.5)	
	3b	130 (9.8)	27 (20.3)		1 (0.1)	0 (0.0)		98 (16.5)	21 (20.0)		31 (49.2)	6 (37.5)		11 (11.8)	16 (40.0)	
M stage	0	1261 (95.2)	117 (88.0)	<0.001	667 (100.0)	12 (100.0)	—	594 (100.0)	105 (100.0)	—	0 (0.0)	0 (0.0)	—	83 (89.2)	34 (85.0)	0.492
	1	63 (4.8)	16 (12.0)		0 (0.0)	0 (0.0)		0 (0.0)	0 (0.0)		63 (100)	16 (100)		10 (10.8)	6 (15.0)	
TNM stage	IA	260 (19.6)	0 (0.0)	<0.001	260 (39.0)	0 (0.0)	0.001	0 (0.0)	0 (0.0)	0.003	0 (0.0)	0 (0.0)	—	0 (0.0)	0 (0.0)	0.139
	IB	145 (11.0)	2 (1.5)		145 (21.7)	2 (16.7)		0 (0.0)	0 (0.0)		0 (0.0)	0 (0.0)		1 (1.1)	1 (2.5)	
	IIA	89 (6.7)	2 (1.5)		89 (13.3)	2 (16.7)		0 (0.0)	0 (0.0)		0 (0.0)	0 (0.0)		2 (2.2)	0 (0.0)	
	IIB	173 (13.1)	8 (6.0)		173 (25.9)	8 (66.7)		0 (0.0)	0 (0.0)		0 (0.0)	0 (0.0)		6 (6.5)	2 (5.0)	
	IIIA	151 (11.4)	15 (11.3)		0 (0.0)	0 (0.0)		151 (25.4)	15 (14.3)		0 (0.0)	0 (0.0)		13 (14.0)	2 (5.0)	
	IIIB	163 (12.3)	25 (18.8)		0 (0.0)	0 (0.0)		163 (27.4)	25 (23.8)		0 (0.0)	0 (0.0)		18 (19.4)	7 (17.5)	
	IIIC	280 (21.1)	65 (48.9)		0 (0.0)	0 (0.0)		280 (47.1)	65 (61.9)		0 (0.0)	0 (0.0)		43 (46.2)	22 (55.0)	
	IV	63 (4.8)	16 (12.0)		0 (0.0)	0 (0.0)		0 (0.0)	0 (0.0)		63 (100)	16 (100)		10 (10.8)	6 (15.0)	

Abbreviations: SD: standard deviation; ENMN: extranodal metastasis negative; ENMP: extranodal metastasis positive.

**Table 2:** Multivariate logistic regression analyses of the relationship between extranodal metastasis with clinicopathological features in different groups of patients in this study

Clinicopathological features	All patients (n=1457)		TNM I-II stage (n=679, 46.6%)		TNM III stage (n=699, 48.0%)		TNM IV stage (n=79, 5.4%)		ENMP (n=133, 9.1%)	
	P value	Odds ratio (95% CI)	P value	Odds ratio (95% CI)	P value	Odds ratio (95% CI)	P value	Odds ratio (95% CI)	P value	Odds ratio (95% CI)
Age							0.001	13.919 (3.063-63.254)		
Gender	0.030	1.635 (1.048-2.552)			0.036	1.745 (1.038-2.934)				
Longitudinal location					0.018	1.363 (1.054-1.763)				
Differentiation grade	0.011	2.789 (1.271-6.120)			0.024	3.313 (1.171-9.371)				
Tumor size	0.001	1.534 (1.188-1.981)			<0.001	1.760 (1.318-2.351)			0.003	2.337 (1.326-4.121)
Vessels/nerves invasion							0.040	4.245 (1.069-16.861)		
T stage	0.001	1.395 (1.138-1.712)	0.001	1.925 (1.287-2.878)						
N stage	<0.001	1.357 (1.152-1.599)								

Abbreviations: CI: confidence interval; ENMP: extranodal metastasis positive.

**Figure 4:**
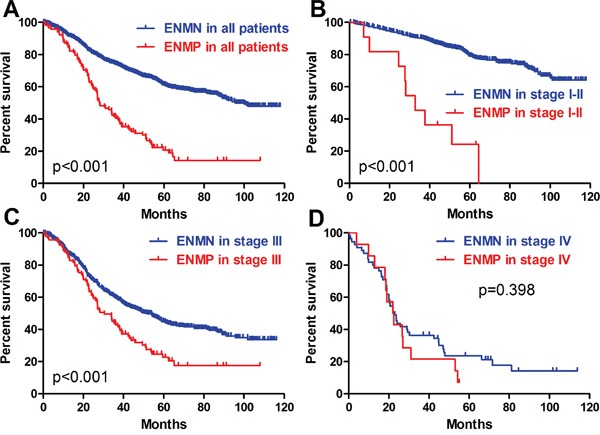
Prognosis of ENMN and ENMP groups in all patients, TNM I-II, III and IV stages

The follow-up rates and median survival time of ENMN group were 91.8% (1216/1324) and 95.7 (0.3-118.0) months, compared with 88.7% (118/133) and 28.0 (0.9-108.0) months in ENMP group. The 3-year survival rates were 73.8% and 40.6% in ENMN and ENMP groups, respectively. In survival analysis, the prognosis of ENMP group was significantly worse than ENMN group in Kaplan-Meier analysis (p<0.001) (Figure 4A). In multivariate analysis through Cox regression, we found that ENM was an independent prognostic factor (p<0.001, hazard ratio (HR) =1.568, 95% confidence interval (CI) [1.243-1.978]), as well as age (p<0.001), tumor size (p<0.001), T stage (p=0.003), N stage (p<0.001) and M stage (p=0.045) (Table 3).

**Table 3:** Survival outcomes of different groups of patients in this study

Features	All patients (n=1457)		TNM I-II stage (n=679, 46.6%)		TNM III stage (n=699, 48.0%)		TNM IV stage (n=79, 5.4%)		ENMP (n=133, 9.1%)	
	ENMN	ENMP	ENMN	ENMP	ENMN	ENMP	ENMN	ENMP	1-2	>2
Patients (n)	1324	133	667	12	594	105	63	16	93	40
Follow-up (n, %)	1216, 91.8%	118, 88.7%	618, 92.7%	11, 91.7%	543, 91.4%	93, 88.6%	55, 87.3%	14, 87.5%	83, 89.2%	35, 87.5%
Range of follow-up time (months)	0.3-118.0	0.9-108.0	0.6-118.0	7.1-64.4	0.3-116.2	0.9-108.0	0.3-113.9	3.9-55.1	0.9-108.0	2.4-72.0
Median follow-up time (months)	66.1	60.9	64.8	63.1	68.3	60.9	84.6	55.1	60.9	45.9
95% confidence interval	[63.6-68.6]	[55.5-66.2]	[61.4-68.2]	[34.5-91.6]	[63.5-73.1]	[55.9-65.8]	[63.4-105.9]	[—]	[56.0-65.7]	[28.1-63.8]
Median survival time (months)	95.7	28.0	[—]	32.7	51.0	30.5	23.0	22.0	34.1	22.0
95% confidence interval	[—]	[21.7-34.3]	[—]	[21.9-43.4]	[43.4-58.6]	[23.4-37.6]	[19.3-26.7]	[15.6-28.4]	[24.3-43.9]	[17.5-26.4]
3-year overall survival rate	73.8%	40.6%	90.0%	45.5%	59.1%	43.9%	36.8%	18.8%	47.0%	25.4%
**Cox regression analyses (p value, hazard ratio, 95% confidence interval)**										
Age	<0.001, 1.354 [1.147-1.597]		0.005, 1.612 [1.152-2.255]		0.002, 1.373 [1.120-1.684]					
Tumor size	<0.001, 1.248 [1.115-1.396]				<0.001, 1.302 [1.126-1.504]					
Cross-sectional location			0.012, 1.121 [1.025-1.226]							
Macroscopic type							0.026, 1.469 [1.048-2.059]			
T stage	0.003, 1.108 [1.035-1.186]				0.002, 1.311 [1.101-1.561]					
N stage	<0.001, 1.446 [1.344-1.556]				<0.001, 1.555 [1.389-1.740]					
M stage	0.045, 1.341 [1.007-1.785]									
Extranodal metastasis	<0.001, 1.568 [1.243-1.978]		<0.001, 5.741 [2.878-11.452]		0.017, 1.381 [1.059-1.802]					
Number of extranodal metastasis									0.012, 1.760 [1.133-2.733]	

Abbreviations: ENMN: extranodal metastasis negative; ENMP: extranodal metastasis positive.

### TNM I-II stages

In TNM I-II stages, we found that there were significantly more old patients (p=0.031) and more tumors with size ≥5cm (p=0.009), advanced T stage (p<0.001) and TNM stage (p<0.001) in ENMP subgroup (n=12, 1.8%) than in ENMN subgroup (n=667, 98.2%) (Table 1), in which only T stage (p=0.001) was analyzed as the independent correlated factor in logistic regression (Table 2). The follow-up rates, median survival time and 3-year survival rates of ENMN and ENMP subgroups were 92.7%, not applicable, 90.0% and 91.7%, 32.7 months, 45.5%, respectively. Up to the end of follow-up time, the survival rate of ENMN subgroup was still higher than 50%, therefore, the median survival time of this subgroup was not applicable. Kaplan-Meier curve showed that ENMP subgroup had remarkably worse prognosis than ENMN subgroup (p<0.001) (Figure 4B). Cox regression revealed that ENM (p<0.001, HR=5.741, 95%CI [2.878-11.452]), age (p=0.005) and tumor size (p=0.012) were the independent prognostic factors in TNM stage I-II (Table 3).

### TNM III stage

**Figure 5:**
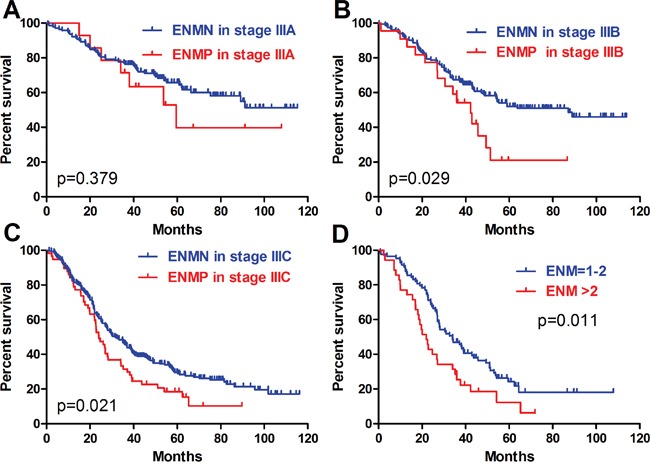
Prognosis of ENMN and ENMP groups in TNM IIIA, IIIB, IIIC stages, and ENM=1-2 and >2

In TNM stage III, ENMP subgroup (n=105, 15.0%) had significantly more male patients and more tumors with UML location (p=0.002), size ≥8cm (p<0.001), poorly differentiation (p=0.013), advanced N stage (p=0.008) and TNM stage (p=0.003) than ENMN subgroup (n=594, 85.0%) (Table 1). Logistic regression showed that gender (p=0.036), longitudinal location (p=0.018), differentiation (p=0.024) and tumor size (p<0.001) were independently correlated to ENM (Table 2). The follow-up rate, median survival time and 3-year survival rate of ENMN subgroup were 91.4%, 51.0 months, 59.1%, compared with 88.6%, 30.5 months, 43.9% of ENMP subgroup, respectively. Kaplan-Meier curve showed that ENMP subgroup had obviously worse survival outcome than ENMN subgroup (p<0.001) (Figure 4C). Cox regression demonstrated that ENM (p=0.017, HR=1.381, 95%CI [1.059-1.802]), age (p=0.002), tumor size (p<0.001), T stage (p=0.002) and N stage (p<0.001) were independently related to prognosis (Table 3). Additionally, we further compared the prognostic difference between ENMN and ENMP patients in TNM IIIA, IIIB and IIIC stages. The results showed that ENMP patients had significantly worse survival outcomes than ENMN patients in stage IIIB (p=0.029) (Figure 5B) and IIIC (p=0.021) (Figure 5C). The difference between ENMN and ENMP patients in IIIA was not significant (p=0.379) (Figure 5A), but the trend was still obvious.

### TNM IV stage

Regarding TNM IV stage, significantly more patients with old age (p<0.001) and distal GC (p=0.001) were found in ENMP subgroup (n=16, 20.3%) than those in ENMN subgroup (n=63, 79.7%) (Table 1). However, no differences were shown in other clinicopathological features. Logistic regression showed that age (p=0.001) and vessel/nerve invasion (p=0.040) were independently associated with ENM (Table 2). The follow-up rates, median survival time and 3-year survival rates of ENMN and ENMP subgroups were respectively 87.3%, 23.0 months, 36.8% versus 89.2%, 22.0 months and 18.8%. Kaplan-Meier curve showed that the difference in prognosis between ENMP and ENMN subgroups was not significant (p=0.398) (Figure 4D). Cox regression revealed that only macroscopic type was the independent prognostic factor (p=0.026, HR=1.469, 95%CI [1.048-2.059]) (Table 3).

### ENMP patients

ENMP patients were subdivided into ENM=1-2 (n=93, 69.9%) and ENM>2 subgroups (n=40, 30.1%). We found that ENM>2 subgroup had significantly more tumors with size ≥8cm (p=0.002) and N3b stage (p=0.016) than ENM=1-2 subgroup (Table 1). Multivariate analysis revealed that tumor size was the independent correlated factor to the number of ENM (p=0.003) (Table 2). The follow-up rate, median survival time and 3-year survival rate of ENM=1-2 subgroup were 89.2%, 34.1 months, 47.0%, compared with 87.5%, 22.0 months, 25.4% of ENM>2 subgroup, respectively. Kaplan-Meier curve showed that the prognosis of ENM>2 subgroup was significantly worse than that of ENM=1-2 subgroup (p=0.011) (Figure 5D). Cox regression revealed that the number of ENM was the only independent prognostic factor (p=0.012, HR=1.760, 95%CI [1.133-2.733]) in ENMP subgroup (Table 3).

### Relationship between ENM and TNM stage

**Figure 6:**
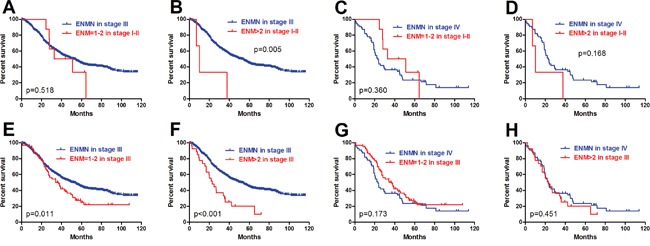
Comparison of ENM=1-2 and >2 in TNM I-III with ENMN patients in TNM III and IV stages

In order to research the relationship between the different number of ENM and TNM stage, we further compared the prognosis of ENM=1-2 and ENM>2 subgroups in ENMP patients with ENMN patients in TNM III and IV stages. To avoid the influence of TNM IV stage, we only included ENMP patients with TNM I-III stages. The results showed that there was no significant difference in survival outcomes between ENM=1-2 in TNM I-II stages and ENMN patients in TNM III (p=0.518) (Figure 6A) or TNM IV stage (p=0.360) (Figure 6C). The prognosis of ENM>2 in TNM I-II stage was significantly worse than ENMN patients in TNM III stage (p=0.005) (Figure 6B), but similar to ENMN patients in TNM IV stage (p=0.168) (Figure 6D). Subsequently, we found that ENM=1-2 (p=0.011) (Figure 6E) and ENM>2 (p<0.001) (Figure 6F) in TNM III stage had remarkably worse prognosis than ENMN patients in TNM III stage. Regarding TNM IV stage, significant prognostic differences were found neither between ENM=1-2 in TNM III stage and ENMN patients in TNM IV stage (p=0.173) (Figure 6G) nor between ENM>2 in TNM III stage and ENMN patients in TNM IV stage (p=0.451) (Figure 6H). Although there was no significant difference between ENM=1-2 subgroup in TNM III stage and ENMN patients in TNM IV stage through log-rank test and Gehan-Breslow-Wilcoxon test (p=0.055), we noticed that the former patients still had a trend with better survival outcome than the latter patients within 60 months. All these results indicated that the prognosis of ENM>2 in TNM I-III stages was similar to ENMN in TNM IV stage.

**Figure 7:**
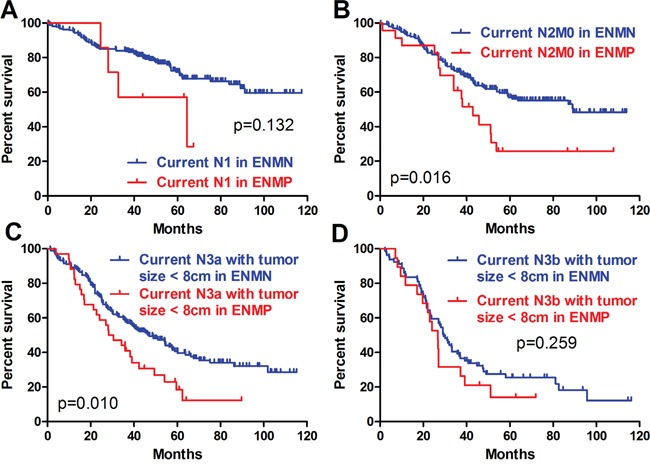
Prognosis of ENMP and ENMN patients in current N stages

Because ENM was currently counted as the metastatic lymph nodes in the N determination according to JGCA, we adopted this recommendation and defined the current N stage as the combination of metastatic lymph nodes and ENM to evaluate the prognostic differences between ENMN and ENMP patients in the same current N stages. Current N0 stage was not analyzed because there was no ENMP patients. In other current N stages, we compared the baselines of current N1, N2, N3a and N3b stages. In order to balance the baseline (like M stage, tumor size), we only compared the differences in current N1 stage, current N2M0 stage, current N3a stage with tumor size <8cm, and current N3b stage with tumor size <8cm (Table 4). Although there were no significant prognostic differences in current N1 stage (p=0.132) (Figure 7A) and current N3b stage with tumor size <8cm (p=0.259) (Figure 7D), the trends of ENMN and ENMP patients in these two current N stages should not be ignored. Kaplan-Meier analyses indicated that the prognostic differences were significant in current N2M0 stage (p=0.016) (Figure 7B) and current N3a stage with tumor size <8cm (p=0.010) (Figure 7C), indicating that it was more reasonable to categorize ENM as an independent factor but not lymph nodes.

**Table 4:** Clinicopathological features of extranodal metastasis negative and positive groups in different current N stages in this study

Clinicopathological features		Current N1 stage (n=238)			Current N2M0 stage (n=233)			Current N3a stage with tumor size <8cm (n=233)			Current N3b stage with tumor size <8cm (n=109)		
		ENMN	ENMP		ENMN	ENMP		ENMN	ENMP		ENMN	ENMP	
		n=231	n=7	P value	n=206	n=27	P value	n=194	n=39	P value	n=88	n=21	P value
Age (years)	≥60	111 (48.1)	5 (71.4)	0.271	88 (42.7)	15 (55.6)	0.207	80 (41.2)	16 (41.0)	0.980	28 (31.8)	8 (38.1)	0.583
	<60	120 (51.9)	2 (28.6)		118 (57.3)	12 (44.4)		114 (58.8)	23 (59.0)		60 (68.2)	13 (61.9)	
Gender	Male	160 (69.3)	5 (71.4)	1.000	148 (71.8)	20 (74.1)	0.808	135 (69.6)	31 (79.5)	0.213	53 (60.2)	14 (66.7)	0.586
	Female	71 (30.7)	2 (28.6)		58 (28.2)	7 (25.9)		59 (30.4)	8 (20.5)		35 (39.8)	7 (33.3)	
Longitudinal location	U	59 (25.5)	4 (57.1)	0.198	59 (28.6)	5 (18.5)	0.589	47 (24.2)	12 (30.8)	0.370	16 (18.2)	1 (4.8)	0.229
	M	42 (18.2)	1 (14.3)		46 (22.3)	6 (22.2)		36 (18.6)	7 (17.9)		15 (17.0)	5 (23.8)	
	L	129 (55.8)	2 (28.6)		99 (48.1)	16 (59.3)		110 (56.7)	19 (48.7)		54 (61.4)	13 (61.9)	
	UML	1 (0.4)	0 (0.0)		2 (1.0)	0 (0.0)		1 (0.5)	1 (2.6)		3 (3.4)	2 (9.5)	
Cross sectional location	Lesser	114 (49.4)	3 (42.9)	0.492	117 (56.8)	18 (66.7)	0.976	96 (49.5)	23 (59.0)	0.576	49 (55.7)	11 (52.4)	0.949
	Greater	19 (8.2)	2 (28.6)		18 (8.7)	2 (7.4)		11 (5.7)	4 (10.3)		7 (8.0)	2 (9.5)	
	Anterior	16 (6.9)	0 (0.0)		8 (3.9)	0 (0.0)		13 (6.7)	1 (2.6)		5 (5.7)	1 (4.8)	
	Posterior	27 (11.7)	1 (0.0)		19 (9.2)	2 (7.4)		17 (8.8)	3 (7.7)		3 (3.4)	0 (0.0)	
	Double	36 (15.6)	1 (14.3)		22 (10.7)	2 (7.4)		24 (12.4)	2 (5.1)		7 (8.0)	1 (4.8)	
	Circumference	19 (8.2)	0 (0.0)		22 (10.7)	3 (11.1)		33 (17.0)	6 (15.4)		17 (19.3)	6 (28.6)	
Macroscopic type	Early stage	30 (13.0)	0 (0.0)	0.307	6 (2.9)	0 (0.0)	0.768	7 (3.6)	2 (5.1)	0.675	2 (2.3)	0 (0.0)	0.260
	Borrmann I	11 (4.8)	1 (14.3)		7 (3.4)	2 (7.4)		5 (2.6)	3 (7.7)		3 (3.4)	0 (0.0)	
	Borrmann II	120 (51.9)	6 (85.7)		99 (48.1)	14 (51.9)		107 (55.2)	18 (46.2)		41 (46.6)	10 (47.6)	
	Borrmann III	64 (27.7)	0 (0.0)		86 (41.7)	9 (33.3)		65 (33.5)	16 (41.0)		35 (39.8)	6 (28.6)	
	Borrmann IV	6 (2.6)	0 (0.0)		8 (3.9)	2 (7.4)		10 (5.2)	0 (0.0)		7 (8.0)	5 (23.8)	
Differentiation grade	Well	3 (1.3)	0 (0.0)	0.552	0 (0.0)	0 (0.0)	0.108	1 (0.5)	0 (0.0)	0.677	0 (0.0)	0 (0.0)	0.107
	Moderately	52 (22.5)	1 (14.3)		31 (15.0)	1 (3.7)		18 (9.3)	3 (7.7)		10 (11.4)	0 (0.0)	
	Poorly	176 (76.2)	6 (85.7)		175 (85.0)	26 (96.3)		175 (90.2)	36 (92.3)		78 (88.6)	21 (100.0)	
Tumor size (cm)	<2.5	30 (13.0)	0 (0.0)	0.296	14 (6.8)	1 (3.7)	0.181	12 (6.2)	0 (0.0)	0.113	3 (3.4)	0 (0.0)	0.059
	2.5-5	105 (45.5)	3 (42.9)		72 (35.0)	8 (29.6)		71 (36.6)	12 (30.8)		28 (31.8)	3 (14.3)	
	5-8	74 (32.0)	3 (42.9)		97 (47.1)	12 (44.4)		111 (57.2)	27 (69.2)		57 (64.8)	18 (85.7)	
	≥8	22 (9.5)	1 (14.3)		23 (11.2)	6 (22.2)		—	—		—	—	
Vessels/nerves invasion	Negative	197 (85.3)	6 (85.7)	0.975	167 (81.1)	20 (74.1)	0.392	134 (69.1)	23 (59.0)	0.221	60 (68.2)	14 (66.7)	0.894
	Positive	34 (14.7)	1 (14.3)		39 (18.9)	7 (25.9)		60 (30.9)	16 (41.0)		28 (31.8)	7 (33.3)	
T stage	1a	19 (8.2)	0 (0.0)	0.104	0 (0.0)	0 (0.0)	0.840	1 (0.5)	0 (0.0)	0.356	0 (0.0)	0 (0.0)	0.411
	1b	37 (16.0)	0 (0.0)		5 (2.4)	0 (0.0)		7 (3.6)	0 (0.0)		1 (1.1)	0 (0.0)	
	2a	21 (9.1)	0 (0.0)		18 (8.7)	1 (3.7)		11 (5.7)	2 (5.1)		0 (0.0)	0 (0.0)	
	2b	26 (11.3)	1 (14.3)		11 (5.3)	2 (7.4)		14 (7.2)	0 (0.0)		3 (3.4)	1 (4.8)	
	3	28 (12.1)	1 (14.3)		30 (14.6)	5 (18.5)		12 (6.2)	4 (10.3)		4 (4.5)	0 (0.0)	
	4a	91 (39.4)	5 (71.4)		130 (63.1)	18 (66.7)		128 (66.0)	29 (74.4)		68 (77.3)	16 (76.2)	
	4b	9 (3.9)	0 (0.0)		12 (5.8)	1 (3.7)		21 (10.8)	4 (10.3)		12 (13.6)	4 (19.0)	
M stage	0	229 (99.1)	7 (100.0)	0.805	206 (100.0)	27 (100.0)	—	179 (92.3)	35 (89.7)	0.600	70 (79.5)	18 (85.7)	0.521
	1	2 (0.9)	0 (0.0)		—	—		15 (7.7)	4 (10.3)		18 (20.5)	3 (14.3)	

Abbreviations: SD: standard deviation; ENMN: extranodal metastasis negative; ENMP: extranodal metastasis positive.

For ENMP patients, we compared the accuracy of prognostic prediction between current TNM stage alone (ENM was counted in N stage) and TNM stage (ENM was not counted in N stage) plus the number of ENM through R software. The results showed that the C-index of current TNM stage alone was 0.611 (95%CI 0.559-0.663), compared with 0.619 (95%CI 0.561-0.677) of TNM stage plus the number of ENM, and the difference was remarkable (p=0.005).

## DISCUSSION

At present, TNM stage including invasion depth, metastasis of lymph nodes and distant metastasis has been considered as the primary factor to predict the prognosis of GC [1]. However, the outcomes of some patients with the same TNM stage might be completely different. Therefore, it is crucially important to find out other ways to increase the predictive accuracy of the prognosis in GC patients. ENM is one of the controversial characteristics in TNM stage. This present study with relative large sample size highlighted the prognostic significance and the role in TNM stage of ENM in GC. The patients in ENMP group had remarkably more advanced tumors and suffered significantly worse prognosis than those in ENMN group in all patients, which was similar to many previous studies [2, 5–7, 10]. Another previous study reported that ENM was correlated with intestinal type [9]. When stratified by TNM stage, the similar results were found in TNM I-II, III, IIIB and IIIC stages, but not in TNM IV stage. In Cox regression analyses, ENM was also demonstrated as an independent prognostic factor in all patients, TNM I-II and III stages. These results indicated that ENM was a good index to distinguish the prognostic differences between ENMN and ENMP in these groups, but not applicable in TNM IV stage.

Furthermore, in order to investigate the significance of the number of ENM, patients with ENMP in our study were subdivided into ENM=1-2 and ENM >2 with the cut-off value of 2, calculated by X-tile software. The results illustrated that ENM>2 subgroup had significantly more lager tumor and worse prognosis than ENM=1-2 subgroup, and demonstrated that the number of ENM could be regarded as an independent prognostic factor in ENMP group, which was consistent with the previous study [3]. Subsequently, we researched the relationship of ENM and TNM stage, because of the controversial role of ENM in TNM stage. Due to the prognostic difference between ENM=1-2 and ENM>2 subgroups and in order to avoid the interrelationship of ENM and TNM IV, we compared these two subgroups in TNM I-III stages with ENMN groups in TNM III and TNM IV stages. The Kaplan-Meier curves showed that the prognosis of ENM>2 in TNM I-III stages was significantly worse than that of ENMN groups in TNM III stage, but very similar to that of TNM IV, indicating that ENM>2 in TNM III stage should be categorized in TNM IV stage. However, the role of ENM=1-2 in TNM I-II stages was still ambiguous and should be further investigated. However, a previous study found that ENM was associated with synchronous distant metastasis [11]. All these results suggested that ENM was closely with TNM IV stage.

From the Japanese classification of GC by JGCA, ENM is currently counted as a metastatic lymph node in the N determination [4]. If this classification is reasonable, the patients with the same current N stage should have similar prognosis. Nevertheless, we found that the prognosis of the patients with ENM in current N2M0 stage and current N3a stage with tumor size <8cm was significantly worse than those without ENM. And the trend of different prognosis in current N1 stage and N3b stage with tumor size <8cm should not be neglected. Based on these results, we thought that it might not be reasonable that ENM should be counted as lymph nodes and categorized in N stage. Some previous studies reported that it should be more suitable for ENM to be treated as a form of serosal invasion [10].

For the patients with ENM, C-index calculated by R software was used to compared the accuracy of prognostic prediction between current TNM stage alone (ENM was counted in N stage) and TNM stage (ENM was not counted in N stage) plus the number of ENM. We found that the latter had a larger C-index than the former, with significant difference. C-index has gradually been applied to compare the accuracy of prognostic prediction by many studies [12–13]. In other study, they used Akaike information criterion, linear trend X^2^, likelihood ratio X^2^ to compare the homogeneity, discriminatory ability and monotonicity of gradients [3, 5]. Nevertheless, we noticed that there were no p values between different groups, indicating that whether the differences were significant was not clear.

The average number of harvested lymph nodes in our study was 27.8. As reported by other previous studies, the average number of harvested lymph nodes was from 14.7 to 34.0 [2–3, 6–7]. As we know, insufficient lymphadenectomy will strongly impact on the accuracy of N stage and the incidence of ENM. To eliminate the influence, we excluded the patients in TNM II-IV stages with harvested lymph nodes less than 15. However, the incidence of ENM was approximately 9% and lower than 13%-14% reported by other previous studies [2–3]. We noticed that the percentage of TNM I stage of one study was obviously lower than that of ours [3]. And some studies only researched ENM in the patients with positive lymph nodes or with esophagogastric cancer, which reported the incidence of ENM between 24.6% and 42.3% [6–7]. The different constitution of included patients might cause the various positive rates of ENM. Other report found that ENM had relationship with peritoneal metastasis [14]. Some studies made a further investigation in the morphology the capsule of ENM, showing that the patients with capsule rupture ENM had significantly worse survival outcomes than those with no capsule rupture ENM [15].

There were some limitations of this retrospective study. This study was from a single institute and the validation of our conclusion should be confirmed through other studies, especially the perspective ones. Although the number of total patients was more than 1000, the number of ENMP was still not large enough. Insufficient patients in some subgroups might influence the statistics and the final results. Retrospective studies might exist some selection bias of patients. However, we tried our best to make a detailed investigation of the prognosis significance of ENM and its role in TNM stage on the basis of multiple comparisons stratified by TNM stage, current N stage and the number and status of ENM.

In conclusion, the patients in ENMP subgroup had more advanced GC and worse prognosis than those in ENMN subgroup. Because of the different prognosis, ENM within regional lymph nodes station might not be counted as metastatic lymph nodes in N stage. And it might be more reasonable to categorize ENM>2 into TNM IV stage.

## MATERIALS AND METHODS

### Patients

In this study, the patients who underwent gastrectomy plus lymphadenectomy with curative intention for primary GC in West China Hospital from January 2005 to December 2011 were retrospectively enrolled. The patients with positive marginal residue were excluded. To reduce the impact of insufficient lymphadenectomy on the prognosis and the positive rate of ENM, we also excluded the patients in stage II-IV with less than 15 lymph nodes harvested (ENM was not counted) in surgery. Finally, a total of 1457 patients were included. The clinicopathological characteristics including age, gender, tumor location, macroscopic type, differentiation grade, tumor size, vessels and nerves invasion and TNM stage according to Japanese classification of GC by JGCA [4] were collected. Follow-up information through telephones, mails and outpatient visit were conducted up to January 2015. The West China Hospital research ethics committee approved this retrospective study.

### Extranodal metastasis

After gastrectomy and lymphadenectomy, the pathological examination of resected specimens was carefully performed under microscope to find the regional lymph nodes. According to JGCA, ENM was defined as the tumor nodule without histological evidence of lymph node structure in the lymphatic drainage area of GC. The number of these ENM was also recorded. Because ENM within regional lymph nodes station was currently counted as a metastatic lymph node in the N determination according to JGCA, we revised the N stage without considering ENM in this present study. Therefore, TNM stage of some patients with ENM was also correspondingly changed. Additionally, in order to research the significance of different number of ENM, we used X-tile software (Version 3.6.1, Yale University) to calculate the optimal cut-point of the number of ENM. And we found that the cut-point was 2. Therefore, the patients with ENM were subsequently subdivided into ENM=1-2 subgroup and ENM>2 subgroup.

### Immunohistochemistry

The tissue slices (4 μm) were deparaffinized with xylene and rehydrated in a graded alcohol series and distilled water. After blocking the endogenous peroxidase with hydrogen peroxide, citrate buffer (ZhongShan Golden Bridge Biotechnology Co., Ltd) was used to perform antigen retrieval in water bath at 95°C for 35 minutes. After naturally cooling down, the slices were incubated with primary monoclonal antibody to EpCAM (1:800, Abcam) at 4°C overnight. Subsequently, these slices were incubated with peroxidase-conjugated polymer (EnVisionTM Detection Kit, Gene Tech (Shanghai) Company Limited) for 30 minutes at room temperature. Finally, the slices were stained with diaminobenzidine chromogen solution (1:50, EnVisionTM Detection Kit, Gene Tech (Shanghai) Company Limited) and counterstained with hematoxylin (ZhongShan Golden Bridge Biotechnology Co., Ltd). Primary antibody incubation was omitted in negative controls. The figures were captured through Axio Imager A2 (Zeiss) and Scope A1 (Zeiss).

### Statistical analyses

Statistical analyses were performed by SPSS software (Version 22, IBM). Unordered categorical variable and ranked data was analyzed through chi-square test and rank sum test (Mann-Whitney U test), respectively. If homogeneity of variance and normal distribution, continuous data was analyzed through Student's t-test. Otherwise, rank sum test was used. Logistic regression was used in multivariate correlation analysis. Kaplan-Meier method and life-table method were used to calculate the cumulative survival rate. Log-rank test and Cox's proportional hazard regression model were conducted for univariate and multivariate survival analyses, respectively. Prism 5 for Windows (Version 5.01, GraphPad Software) was used to draft the figure of Kaplan-Meier curve. Comparisons of accuracy of prognostic prediction between different models were performed with the package of Harrell Miscellaneous (Hmisc) and Regression Modeling Strategies (rms) in R for Windows (Version 3.2.0, R Foundation for Statistical Computing) and was evaluated by the C-index, with the meaning of that the larger the C-index, the more accurate was the prognostic prediction. Two-sided P value less than 0.05 was considered as statistical significance.
